# Biochemical Composition, Antioxidant Activity and Antiproliferative Effects of Different Processed Garlic Products

**DOI:** 10.3390/molecules28020804

**Published:** 2023-01-13

**Authors:** Jingyang Lu, Nannan Li, Shuqin Li, Wei Liu, Mingyue Li, Min Zhang, Haixia Chen

**Affiliations:** 1 Tianjin Key Laboratory for Modern Drug Delivery & High-Efficiency, School of Pharmaceutical Science and Technology, Tianjin University, Tianjin 300072, China; 2 State Key Laboratory of Nutrition and Safety, Tianjin Agricultural University, Tianjin 300384, China; 3 State Key Laboratory of Nutrition and Safety, Tianjin University of Science & Technology, Tianjin 300457, China

**Keywords:** antioxidant activity, antiproliferative effect, constituents, different processed garlic, gas chromatography-mass spectrometry (GC-MS), organosulfur compounds, principal components analysis (PCA)

## Abstract

Garlic (*Allium sativum* L.) is a type of agricultural product that is widely used as a food spice, herb and traditional medicine. White garlic (WG) can be processed into several kinds of products, such as green garlic (GG), Laba garlic (LAG) and black garlic (BG), which have multiple health effects. In this study, GC-MS (gas chromatography-mass spectrometry), DPPH (1,1′-diphenyl-2-propionyl hydrazide) radical scavenging, hydroxyl radical scavenging and MTT (3-(4,5-dimethylthiazol-2-yl)-2,5-diphenyl tetrazolium bromide) in vitro assays were used to compare the composition, antioxidant and antiproliferation effects of different processed garlic extracts. The relationship between the constituents and the bioactivities was analyzed using the principal components analysis (PCA) and heatmap analysis. BG showed the highest antioxidant activity (IC_50_ = 0.63 ± 0.02 mg/mL) in DPPH radical assays and the highest antioxidant activity (IC_50_ = 0.80 ± 0.01 mg/mL) by hydroxyl radical assay. Moreover, GC-MS results showed that 12 organosulfur compounds were detected in the extracts of four garlic products, and allyl methyl trisulfide showed a positive relation with the anticancer activity on SMMC-7721 cells (hepatocellular carcinoma cells). The results suggested that the processing of garlic had a significant influence on the constituents and antioxidant effects and that GG, LAG and BG might be better candidates for the related functional food products compared to WG.

## 1. Introduction

Garlic (*Allium sativum* L.) is widely cultivated all over the world and is used not only in cuisines but has been used as a herbal medicine for centuries [1]. Research indicates that garlic has potential health benefits such as antioxidant, hyperglycemic, antibacterial, anti-inflammatory, anti-atherosclerotic and anticancer capacities [2,3]. Garlic also comprises an abundance of nutritional compounds such as amino acids, fructose and phenols, and has a high concentration of organosulfur compounds. These organosulfur compounds include lipid and water-soluble sulfur compounds such as alliin, A-joene, 1,2-vinyldithiin, diallyl sulfide (DAS), diallyl disulfide (DATS) and S-allyl cysteine (SAC) [4], which show anti-cancer effects against colon cancer and gastric cancer. It also contains various phenolic compounds with antioxidant capacities, such as caffeic acid, myricetin and *p*-coumaric acid [5]. However, the chemical composition and bioactive compounds are always influenced by the variety, location, storage condition and processing treatment [6,7,8].

With the increasing consumption of garlic, fresh garlics were processed into various kinds of products including black garlic (BG), green garlic (GG) and Laba garlic (LAG), using thermal processing or acid, which have distinctive flavors, colors and contain a variety of pharmacological benefits. Laba garlic has a long history of use as a traditional food, especially in the north of China. Green garlic with a blue or green color is similar to Laba garlic, with differences in processing. Studies have shown that the formation of characteristic flavor was mainly influenced by the decreased content of organosulfur compounds, such as diallyl disulfide, diallyl tetrasulphide, 3-vinyl-1,2-dithiacyclohex-4-ene and so on, and the increased content of non-organosulfur compounds, including acetic acid, butanoic acid, and benzoic acid in the soaking processing [7]. Black garlic with a less pungent flavor and excellent activity is processed at a relatively high temperature and humidity environment [9]. Previous studies have shown that there were differences in the antioxidant activity and components between black garlic and fresh garlic. It was reported that the antioxidant capacity of black garlic was stronger, the content of ascorbic acid decreased, the content of sugar and polyphenols increased, and the composition and content of phenolic acids and flavonoids were different from those of fresh garlic [10]. Furthermore, a metabolomics analysis with headspace-GC-MS revealed significant differences in the volatile profiles of black and fresh garlic. Fresh garlic contained a high concentration of sulfur volatiles, while the fermentation process of black garlic reduced the concentration of sulfur volatiles, and increased the content of sweet and baking volatiles, especially furfural and its derivatives [11]. Hence, different processing methods demonstrate the different formation and accumulation of the bioactive constituents. However, the compositional difference and the effects on the bioactivities of the four types of garlic products (WG, GG, BG and LAG) are still unknown. Based on the previous studies, we assumed that processed garlics might have a better antioxidant activity and anti-proliferation ability. Compared with white garlic, they have better potentials as a functional food and are good candidate products.

In this study, the differences and relationships of the components of four kinds of garlic, including WG, GG, BG and LAG were investigated to explore antioxidant properties and antiproliferative capacities. The relationships between the components and the activities were also discussed using a multivariate statistical analysis. This study can help us understand whether the processing methods cause nutrient loss and reduce the of activity of garlic, which can provide a higher reference value for producers and consumers.

## 2. Results and Discussion

### 2.1. Contents of Total Sugar, Uronic Acid and Protein

The contents of total sugar, uronic acid, protein and monosaccharide composition are presented in Table 1. Different kinds of garlic products played a significant role in the contents of uronic acid and total sugar (*p* < 0.01). The content of total sugar in BG, GG and LAG was 16.80 mg/g, 10.61 mg/g and 7.89 mg/g dried weight (DW), respectively. The content of uronic acid was 3.91 mg/g, 2.28 mg/g and 1.70 mg/g DW. The total sugar and uronic acid in WG were significantly lower than those in BG and GG (*p* < 0.01). The results are in accordance with the previous investigation, that found black garlic to have a higher content of total sugar, total polyphenol and total flavonoids compared with the contents of fresh garlic [12]. Some studies reported that thermal treatment played a more important role than enzymatic hydrolysis in the degradation of polysaccharides [13].

The monosaccharides composition of the total sugars in WG, GG, BG and LAG included fructose, arabinose, xylose, mannose, galactose and glucose, respectively. The glucose content should be set as the standard. Take the molar ratio of fructose to glucose in WG as an example. The molar percentage of fructose is 11.23% and glucose is 53.48%. Then, the molar ratio of fructose:glucose = 11.23:53.48 = 0.21:1.00. Therefore, the molar ratios of monosaccharide compositions in the above four kinds of garlic are 0.21:0.05:0.11:0.36:0.14:1.00, 1.27:0.23:0.21:0.04:7.34:1.00, 0.74:1.85:0.59:0.31:8.5:1.00 and 0.08:0.12:0.11:0.03:0.5:1.00, for fructose, arabinose, xylose, mannose, galactose and glucose, respectively (Table 1). Fructans, which provide the majority of carbohydrates in fresh garlic, are not stable under acidic environments [14]. As the black garlic was processed at a high temperature for a long time, the heat treatment made the polysaccharide degraded, so the glucose and fructose could participate in the Maillard reaction [15], resulting in the color change in the black garlic. This might be related to the lower proportion of glucose and fructose content in BG compared with WG. At the same time, since fructose is a ketose, which is less reactive than glucose [15], compared with WG the proportion of the fructose content in BG was not much lower than that of glucose. The processing time of green garlic at a high temperature was not as long as that of black garlic, which might be related to the fact that the proportion of the glucose content in green garlic was slightly higher than the glucose content in black garlic. The protein content in the garlic extracts of products ranged from 60.03% to 77.32%, depending on the kinds of garlic products. The highest protein content was found in BG (77.32%), and the lowest was found in WG (60.03%) (Table 1). Similarly, previous studies have shown that the protein content is 6.8–7.5% for white garlic, and 6.3–14.5% for black garlic. [16]. The protein content in BG was slightly higher than the protein content in WG.

### 2.2. Total Phenolic and Total Flavonoids Contents in Garlic

The total phenolic content in WG, GG, BG and LAG were 1.25 ± 0.11 mg/g, 1.38 ± 0.07 mg/g, 13.62 ± 0.91 mg/g and 1.56 ± 0.07 mg/g (mg GAE/g extracts), respectively (Table 1). By contrast, the total phenolic content in BG was significantly higher than that of the other three garlic varieties (*p* < 0.01), while the total phenolic contents in WG, GG and LAG had little difference, and the content in LAG was slightly higher. The total phenolic content in BG was about 11 times higher than that in WG. Previous studies have shown that, compared with raw garlic, the total phenolic content in black garlic increased by about seven times [17], which is similar to the results of this study. At the same time, the total flavonoid contents of WG, GG, BG and LAG were 0.32 ± 0.03 mg/g, 0.34 ± 0.04 mg/g, 0.74 ± 0.08 mg/g and 0.45 ± 0.06 mg/g (mg QE/g extracts), respectively, and the total flavonoid content of WG was relatively low (Table 1), which was similar to the total phenolic content. Kim J. S. et al. researched that the total flavonoid content in BG increased by about 1.1–1.3 times compared with fresh garlic [18]. This is also similar to the results of our study. According to 3.2, both GG and BG were heated to 80 °C during processing. BG was heated at 80 °C for 22 days and GG was only soaked at 80 °C for 30 min, while WG and LAG were only kept at about 4 °C during processing. During high-temperature processing, polyphenols and flavonoids were released from the cell matrix, leading to a significantly improved extractability [19]. The long heat treatment at 80 °C made the phenolics and flavonoids in the BG fully release from the cell matrix, which greatly improved the extraction amount. BG was considered as the natural source of inulin and flavonoids that had important biological activity [20]. During fermentation, the conjugated form of phenolic compounds may be converted into a free form under acidic conditions, which enhances their extractability [21]. LAG was soaked in vinegar for 22 days during the processing, and the phenolic compounds changed into a free state under acidic conditions, so the total phenolic content of LAG was higher than that of WG. The GG was soaked in acidic conditions and a high temperature when processing, but its total phenolic and flavonoids contents were between those of WG and LAG, respectively. This might be because the soaking time was too short, so the phenolics and flavonoids could not be fully released from the cell matrix or the conjugated form of phenolics could not be fully converted into a free form. Therefore, we speculate that the change in the total phenolic content depends on the processing method, and that the possible order of the influence of the processing conditions on the total phenolic content and the total flavonoid content in garlic is as follows: high temperature, soaking time and acidic environment.

### 2.3. Total Organosulfur Compounds Content in Garlic

The contents of organosulfur compounds are presented in Table 1. It can be seen that the amounts of organosulfur compounds varied widely in the garlic extracts, ranging from 0.89% to 2.41% (*p* < 0.01). Since thermal processing will tend to reduce the original contents of organosulfur compounds [22], the lowest levels were detected from black garlic, whereas the highest levels were detected from green garlic.

It was reported that the content of water-soluble SAC in black garlic increased because of the hydrolysis of γ-glutamyl-*S*-allyl-L-cysteine (GSAC) and the reduction of alliin during high-temperature processing [23]. A high temperature and too long processing will lead to the volatilization of organosulfur compounds. The contents of organosulfur compounds detected in BG was lower than WG. Combined with the results of GC-MS (Table 2), it can be inferred that more volatile sulfides are produced during the process. During garlic pickling, cytoplasm and cell wall separation and membrane rupture in garlic tissues speeds up the reaction of alliinase and alliin, and the formation of other organosulfur compounds are further accelerated [24]. The contents of organosulfur compounds in LAG and GG increased compared with WG. As the processing time of LAG was too long and the organic sulfide volatilized, the detectable content of LAG was less than that of GG. A short time of high temperature and acid solution immersion might be the possible reasons for the highest content of organosulfur compounds in GG among the four garlic varieties.

### 2.4. GC-MS Analysis

A total of 18 compounds (Table 2) were identified by GC-MS in the n-hexane fraction of WG, GG, BG and LAG, including organosulfur compounds and others. Variation among the garlic products in their volatile sulfur compounds and their compositions had been reported, and 1-propenyl and methyl groups were commonly found in garlic and black garlic [12]. In this study, allyl methyl trisulfide was the dominant compound observed in garlic products, and the content of allyl methyl trisulfide in BG was relatively high. Sulfides, including diallyl disulfide, diallyl trisulfide and 1-Propene-1,1′-thiobis were detected in all four garlic products. Among these compounds, the content of diallyl disulfide in GG was relatively high (2.36%), whereas the content of diallyl trisulfide in LAG was higher (6.10%). Meanwhile, the contents of 1-Propene-1,1′-thiobis in GG and LAG were relatively high (3.82% and 3.65%, respectively). However, allyl methyl sulfide and diallyl tetrasulfide were not found in BG, which might be due to the high-temperature reaction. As reported by Tamaki et al. [25], high-molecular sulfur compounds existed in both raw garlic and heated garlic, and the inactivation of alliinase at a high temperature inhibited the formation of some sulfur compounds. We speculated that this might be the reason why the contents of 1-Propene-1,1′-thiobis and diallyl trisulfide in raw garlic were higher than those in BG. Some compounds such as 1,2-Dithiin-3-ethenyl-3,6-dihydro and ethyl hexadecanoate, were found only in LAG and GG, which might be related to the vinegar soaking during the processing. The odor differences might depend on the type and contents of these organosulfur compounds of the different processed garlics.

### 2.5. The Antioxidant Activity of Ethanol Extracts of the Four Garlic Products

**Figure 1:**
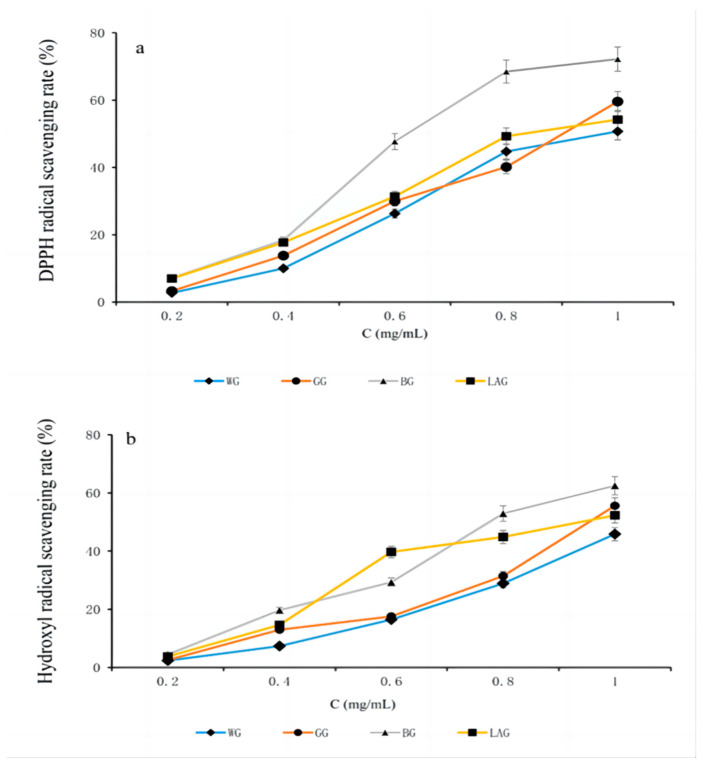
Antioxidant activities of WG, GG, BG and LAG. (**a**) DPPH scavenging activities; (**b**) hydroxyl radical scavenging activities. WG: white garlic; GG: green garlic; BG: black garlic; LAG: Laba garlic; and DPPH: 1,1′-diphenyl-2-picrylhydrazyl.

The antioxidant capacity was measured by DPPH and hydroxyl radical assays, which showed dose-dependent relationships (Figure 1). Among the four types of processed garlic, BG extract showed the highest antioxidant activity (IC_50_ = 0.63 ± 0.02 mg/mL) in DPPH radical assays and the highest antioxidant activity (IC_50_ = 0.80 ± 0.01 mg/mL) by hydroxyl radical assay. The second highest antioxidant activity by DPPH assay and the hydroxyl radical scavenging assay were shown by LAG extract, with the IC_50_ values of 0.87 ± 0.02 mg/mL and 0.88 ± 0.02 mg/mL, respectively. GG showed slender antioxidant activity, which was significantly lower than BG (*p* < 0.01), with IC_50_ values of 0.87 ± 0.02 mg/mL in DPPH radical assays and 1.00 ± 0.03 mg/mL in hydroxyl radical assay. Additionally, compared with BG and LAG, white garlic extract exhibited a lower antioxidant capacity (*p* < 0.01), in which the IC_50_ values were 0.94 ± 0.02 mg/mL and 1.12 ± 0.02 mg/mL, respectively. In the positive control group, the IC_50_ value of ascorbic acid was 97.73 ± 2.35 μg/mL, and the IC_50_ value of BHA was 1.04 ± 0.04 mg/mL. GG, BG, and LAG showed better antioxidant activity than WG and BHA, which indicates that the antioxidant activities were improved after processing. BG ethanol extracts had stronger scavenging activities than WG, which might account for the higher content of phenols and flavonoids [26].

### 2.6. Antiproliferative Activity

To compare the anticancer effects of the four types of garlic, the four n-hexane extracts of WG, GG, BG and LAG on the growth of human cervical cancer cells (HeLa), human ovarian cancer cells (SKOV3) and hepatocellular carcinoma cells (SMMC-7721), 3-(4,5-dimethylthiazol-2-yl)-2,5-diphenyl tetrazolium bromide (MTT) assays were performed. The antiproliferative activity of the four extracts on HeLa cells was in a concentration-dependent manner (Table 3). HeLa cell growth was moderately inhibited by the n-hexane extracts of WG, GG, BG and LAG, with the IC_50_ values of 72.51 ± 3.00 µg/mL, 70.40 ± 3.95 µg/mL, 60.03 ± 6.79 µg/mL, 88.57 ± 3.81 µg/mL, respectively. The inhibition rate of paclitaxel (50 μg/mL) on HeLa cells was 52.34%. Different processing garlic extracts showed a significantly inhibitory difference (*p* < 0.05) at the concentration of 60 µg/mL. The results suggested that the n-hexane extract of BG has potential anticancer activities against cervical carcinoma in vitro. Consistent with our results, it was reported that extracts of garlic showed strong anticancer activities against cervical carcinoma [17].

SKOV3 cells exhibited a dose-dependent sensitivity of the n-hexane extracts of WG, GG, BG and LAG, with IC_50_ values at 72 h around 74.04 ± 4.50 µg/mL, 82.56 ± 2.87 µg/mL, 78.83 ± 2.86 µg/mL, 81.80 ± 4.40 µg/mL, respectively (Table 3), while the inhibition rate of 50 μg/mL paclitaxel was 80.37%. In contrast, the n-hexane extracts of the four garlic varieties had a lower inhibition on the SKOV3 cells. The inhibition of different processing garlic extracts was significantly different (*p* < 0.05) at the concentration of 40 µg/mL. It was reported that S-allyl cysteine had an anti-proliferative capacity on A2780 cells in both dose and time-dependent manners with the IC_50_ of 25 mmol/L [27], which might be one of the main active components in garlic n-hexane extracts that inhibited SKOV3 cells. The effects of the n-hexane extracts of WG, GG, BG and LAG on the growth of SMMC-7721 cells were shown in Table 3. A statistically significant decrease in the number of viable cells at different concentrations of the extracts (from 20 µg/mL to 100 µg/mL) was observed after 72 h treatment. The IC_50_ values of n-hexane extract of WG, GG, BG and LAG ranged from 28.82 ± 1.53 µg/mL to 78.03 ± 4.91 µg/mL. The inhibitions of different processing garlic extracts were significantly different (*p* < 0.05) when the concentration was set at 60 µg/mL or 80 µg/mL. The n-hexane extracts of WG showed weak antiproliferative activity (IC_50_ = 78.03 ± 4.91 µg/mL), while the n-hexane extracts of BG and LAG showed moderate cytotoxicity (28.82 ± 1.53 µg/mL and 35.63 ± 1.97 µg/mL, respectively). The inhibition rate of 50 μg/mL paclitaxel on SMMC-7721 cells was 77.9%. It was reported that diallyl sulfide (DAS) can inhibit liver cancer by inhibition of the formation of DNA adducts in male Sprague–Dawley rats [28]. So, DAS might be one of the main active components in garlic extracts that inhibited the growth of SMMC-7721 cells, which will need further study.

Garlic compounds have been widely received as anticancer products and their anticancer activities are extensively reported in many cancer cell lines. Both water-soluble and lipid-soluble allyl sulfur compounds of garlic exhibited observable chemical properties and excellent antiproliferative effects against various carcinoma cells [29]. The organosulfur compounds are transformed in the processing; alliin is changed into allyl thiosulfinates including diallyl sulfide (DAS), diallyl disulfide (DADS) and A-joene. Several studies have suggested that DAS, DADS and diallyl trisulfide (DATS) inhibit migration and invasion of human colon cancer cells through the inhibition of matrix metalloproteinase expression [30]. After processing, the compositions and contents of the organosulfur compounds in garlic extracts were different, which resulted in different antiproliferative activities.

### 2.7. Multivariate Statistical Analysis

**Figure 2:**
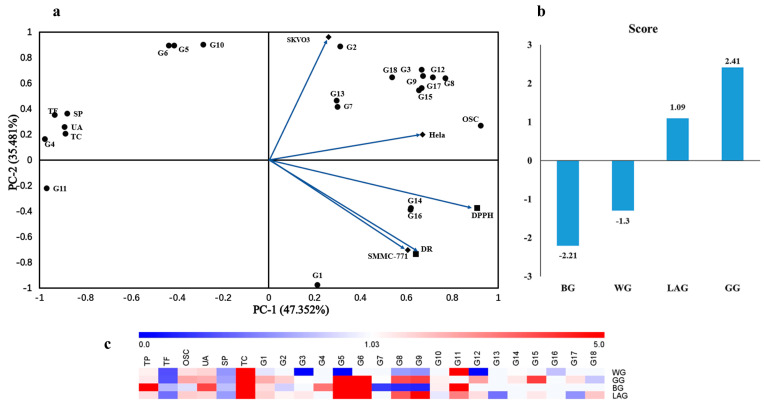
Results of PCA and heatmap analysis (**a**) The component described by two principal components (PC-1) = 47.362% and PC-2 = 35.481% (**b**) The score plots of four kinds of garlic. (**c**) The heatmap correlation of major chemical ingredients of four kinds of garlics.G1: Diallyl disulfide; G2: 2-Hexylthiophene; G3: Allyl methyl sulfide; G4: 1,3,5-trithiane; G5: Allyl-methyl trisulfide; G6: 2-tert-butylhiophenol; G7: Butythiobenzene; G8:1-propene-1,1′-thiobis; G9: Diallyl trisulfide; G10: 3-Acetyl-2,6-heptanedione; G11: Ethyl 4-t-butylbenziate; G12: Diallyl tetrasulphide; G13: 2,5-dimethyl thiophene; G14: Acetyl valeryl; G15: 1,2-Dithiin-3-ethenyl-3,6-dihydro; G16: 10-nonadecanol; G17: Ethyl hexadecanoate; G18: (Z, Z, Z)-9, 12, 15-Octade-catrienoyl ethyl ester; WG: white garlic; GG: green garlic; BG: black garlic; and LAG: Laba garlic.

A principal components analysis (PCA) and heatmap were used to reveal the differences among these samples. To efficiently visualize the dissimilarities of the four kinds of garlic, a PCA was carried out based on the contents of the total sugar, uronic acid (UA), protein and organosulfur compounds, total phenolics, total flavonoids and the results of GC-MS. The antioxidant activity and antiproliferative activity were expressed by IC_50_ in the PCA. According to the results, the first three main components were obtained and they explained 100% of the data variation, of which the principal component 1 (PC-1) contributed 47.352% by most data variation. The principal component 2 (PC-2) explained up to 35.481% of components and the principal component 3 (PC-3) referenced 17.167%. Table 4 shows the factor loadings obtained after varimax rotation. The contribution of a particular variable to its PC (principal component) will be valued according to the absolute value of the loading. The important variables in PC-1 were DPPH radical scavenging activity and the contents of TP (total phenolics), TF (total flavonoids), OSC (organosulfur compounds), which demonstrated that the antioxidant activity might be positively correlated with the total phenolics and flavonoids. The hydroxyl radical scavenging activity and antiproliferative activity in PC-2 might be positively correlated with 2-hexylthiophene, 3-acetyl-2,6-heptanedione, 2-tert-butylhiophenol and the organosulfur compounds such as AMTS (allyl methyl trisulfide) and DADS. Figure 2a shows the biplot of PC-1 and PC-2, Figure 2b shows the score plots of WG, GG, LAG and BG. The types of constituents of BG and WG were less than GG and LAG in the results of GC-MS (Table 2), so BG and WG scored lower.

In order to visualize the contents of the constituents of the four kind garlics and evaluate the correlation, a heatmap was applied for analysis. In this heatmap (Figure 2c), the concentration of compounds was reflected in the colored cells, and the concentration was represented by blue to red from low to high, respectively. The results indicated that the relative contents of compounds were different in each sample, especially regarding the total phenolics and organosulfur compounds. GG and LAG could gather one group, which indicated they had a high similarity.

## 3. Materials and Methods

### 3.1. Chemicals and Reagents

1,1′-diphenyl-2-picrylhydrazyl (DPPH, 97%), 2-Deoxy-D-Ribose (97%), gallic acid (purity ≥ 97.5%) and quercetin (purity ≥ 95%) were provided by Sigma Chemical Co. (St. Louis, MO, USA). Folin–Ciocalteu reagent (FC reagent, purity ≥ 99.0%) and deoxyribose (99.8%) were purchased from Guangfu Fine Chemical Research Institute (Tianjin, China). All other chemicals and reagents were purchased locally.

### 3.2. Processing of Garlic by Different Methods

**Figure 3:**
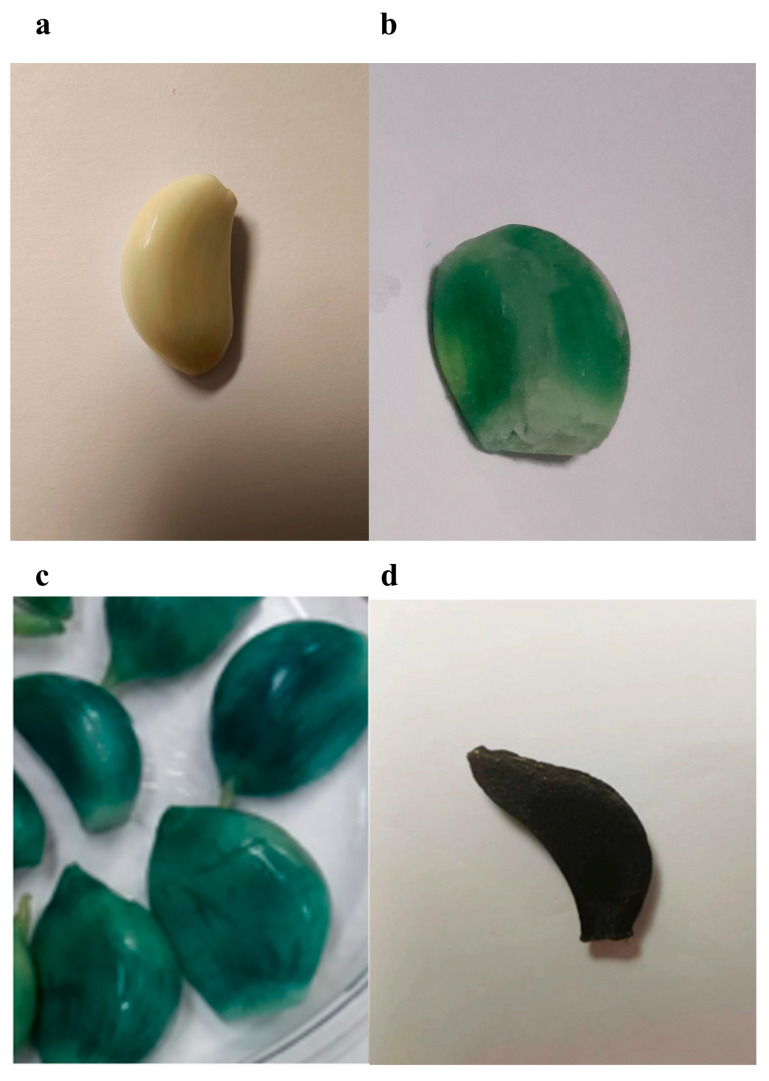
Pictures of four kinds of garlic. (**a**) White garlic, (**b**) green garlic, (**c**) Laba garlic and (**d**) black garlic.

Fresh garlic was purchased from Laiwu City, Shandong Province, China and then stored at 4–6 °C for one month, after which it was considered to be white garlic (WG, Figure 3a). Green garlic (GG, Figure 3b) was prepared according to previous study, garlic was crushed and kept for 30 min at room temperature, soaked in 5% acetic acid at 50 °C for 60 min, then at 80 °C for 30 min [31]. Laba garlic (LAG, Figure 3c) was manufactured following the traditional folk method, with the garlic soaked in vinegar ratio of 1:3 for two weeks at 4 °C. Black garlic (BG, Figure 3d) was processed according to the previous studies [32] with minor modification. Briefly, fresh garlic was heated at 80 °C for 22 days with 80% humidity.

### 3.3. Preparation of Lipid-Soluble Extracts and Water-Soluble Extracts of Different Garlic

Garlic products were peeled and crushed. Each type of garlic (100 g) was extracted by heating and refluxing with 300 mL 80% ethanol at 80 °C for 2 h. Then, the extracts were filtered, the filtrate was collected, and the above operation was repeated twice to extract the filter residue. The ethanol extracts obtained by three instances of filtration were collected and concentrated with a rotary evaporator at 45 °C under vacuum. Then, the ethanol extracts were extracted with the same amount of n-hexane. The antioxidant activity was analyzed and determined.

The garlic residues extracted with ethanol were collected and extracted three times with distilled water (1:4 *w*/*v*) by heating and refluxing at 80 °C for sugar, protein and other analysis.

### 3.4. Determination of the Total Sugar, Uronic Acid, Protein and Monosaccharide Composition

Distilled water extracts were used for the determination of total sugar, uronic acid, protein and monosaccharide composition. The total sugar content was determined by the phenol-sulfuric acid analysis method [33]. The standard curve was drawn with glucose (purity ≥ 99.5%, 0, 50, 75, 100, 125 and 150 μg/mL) as the standard sample, and the total sugar content was calculated according to the standard curve (y = 0.0058x + 0.0141, R^2^ = 0.9960). The glucose concentrations showed a good linear relationship with the absorbance in the range of 0–150 μg/mL. Uronic acid was determined using m-hydroxydipheny method [34]. The standard curve was drawn using D-galactouronic acid (purity ≥ 98%, 0, 32, 64, 96, 128, 160 and 200 μg/mL) as the standard, and the content of uronic acid was calculated according to the standard curve (y = 0.0062x − 0.016 and R^2^ = 0.9958). The D-galactouronic acid concentrations showed a good linear relationship with the absorbance in the range of 0–200 μg/mL. Protein content was analyzed by Coomassie brilliant blue method from a slight amendment of Ma’ s description [35]. The standard curve was drawn with bovine serum protein (purity ≥ 98%, 0, 31.25, 62.5, 125, 250, 500, 1000, 2000 μg/mL.) as the standard sample, and the protein content was calculated according to the standard curve (y = 0.0009x + 0.1251, R^2^ = 0.9968). The concentrations showed a good linear relationship with the absorbance in the range of 0–2000 μg/mL. Gas chromatography (GC, Techcomp GC-7900, Shanghai, China) was applied to measure the monosaccharide composition with a column (30 m × 0.32 mm × 0.5 μm) [36].

### 3.5. Determination of Total Phenolics Contents and Total Flavonoids Contents

N-hexane extracts were used for the determination of total phenolic and flavonoid content. Assay of total phenolic content (TPC) of garlic extracts was determined in accordance with Folin–Ciocalteu’s method [37], and the results were expressed as mg of gallic acid equivalents for per g of extract (mg GAE/g extract). Gallic acid was used with different content (0, 8, 16, 24 and 32 μg/mL) as the standard to draw the standard curve and calculate the total phenolic content from the standard curve (y = 0.0081x + 0.0625, R^2^ = 0.9904). The gallic acid concentrations showed a good linear relationship with the absorbance in the range of 0–32 μg/mL. The total flavonoid content (TFC) was assayed by the AlCl_3_ colorimetric method [38]. Total flavonoid content was expressed as milligram of quercetin equivalents (mg QE/g extract). The standard curve was drawn with quercetin of different contents (0, 8, 16, 32, 48, 64 and 80 μg/mL) as the standard sample, and the total phenolic content was calculated from the standard curve (y = 0.0101x − 0.0079, R^2^ = 0.9962). The quercetin concentrations showed a good linear relationship with the absorbance in the range of 0–80 μg/mL.

### 3.6. Determination of Organosulfur Compounds

N-hexane extracts were used for the determination of organosulfur compounds. The contents of organosulfur compounds in garlic were determined according to Lawson’s method [39], using cysteine as the standard. The standard curve (y = 4.0859x − 0.0098, R^2^ = 0.9997) was established by the homocysteine (purity ≥ 98%, 0, 0.05, 0.10, 0.15, 0.20 and 0.25 mmol/L). The homocysteine concentrations showed a good linear relationship with the absorbance in the range of 0–0.25 mmol/L.

### 3.7. GC-MS Analysis

N-hexane extracts of WG, GG, BG and LAG were used for GC-MS analysis. Determination of the volatile compounds of garlics was performed on a GCMS-QP2010-ES (Shimadzu Japan). The capillary column used was an Agilent HP-5 capillary column (30 m × 0.32 mm I.D., 0.25 μm). The mass selective detector was performed in full-scan mode and the ion source and quadrupole were maintained at 230 °C and 150 °C, respectively. The helium was used as a carrier gas with a flow rate of 2 mL/min and the injector temperature was maintained at 250 °C. The temperature of column oven was initially held constantly as follows: 50 °C for 1 min, then increased to 220 °C at 5 °C/min and held for 2 min. The total run time was approximately 18 min. Samples diluted with n-hexane were injected with volume of 1.0 µL. The mass analyzer was operated constantly with a full scan mode.

Each compound was identified by matching mass spectra and retention indices (RI) with NIST mass spectra libraries and literature data.

### 3.8. Analysis of Antioxidant Activities

N-hexane extracts of WG, GG, BG and LAG were used for the analysis of antioxidant activities, including DPPH radical scavenging activity and hydroxyl radical scavenging activity.

Assay of DPPH free radical scavenging activity: The scavenging effects of the garlic extracts on DPPH radicals were measured according to described method [35]. Results assessed as follows:

Inhibition (%) = [(A_blank_ − A_sample_)/A_blank_] × 100%,

where A_sample_ and A_blank_ were the UV absorbance of the sample and blank, respectively. Ascorbic acid (purity ≥ 99%) was used as the positive control.

Assay of hydroxyl radical scavenging activity: The inhibitory activity of the garlic extracts against hydroxyl radicals was quantified according to described method [40]. Butyl hydroxyanisole (BHA, purity ≥ 98.5%) was used as positive control. The inhibition rate formula was the same as that of DPPH.

### 3.9. Antiproliferative Activity

Human ovarian cancer cells (SKOV3), human cervical cancer cells (HeLa) and hepatocellular carcinoma cells (SMMC-7721) were obtained from Chinese Academy of Sciences Committee on Type Culture Collection Cell Bank (Shanghai, China). All these cells were cultured at 37 °C in a 5% (*v*/*v*) CO_2_ atmosphere.

N-hexane extracts of WG, GG, BG and LAG were used for the analysis of antiproliferative. Cell inhibitory activity was assayed by MTT assay. Briefly, cancer cells were plated into 96-well plates at a density of 1 × 10^5^ cells/mL. After all-night growth, cells were pretreated with a series of concentrations of n-hexane extracts (20, 40, 60, 80 and 100 μg/mL) for 24 h, with blank DMEM medium (Dulbecco’s Modified Eagle Medium) used as the negative control group and 50 μg/mL paclitaxel (purity ≥ 98%) as the positive control. After pretreatment, 20 µL of MTT (purity ≥ 98%, 5 mg/mL) was added to each well, with the cells incubated for a further 4 h and then discarded the supernatant and added 150 µL DMSO (dimethyl sulfoxide, purity ≥ 99.5%) to dissolve the precipitate. The absorbance at 492 nm was measured by Multiskan Spectrum (Perlong, Beijing, China). Inhibitory rate of cells was calculated as follows:

Inhibitory rate = (A_control_ − A_treated_)/A_control_ × 100%.

All assays were conducted with six parallels samples. The IC_50_ value was expressed as the concentration of the extracts that was required for 50% inhibition rate relative to controls.

### 3.10. Statistical Analysis

All measurements were performed in triplicates and the values were expressed as the means ± standard deviation (SD). The SPSS software (SPSS Inc., Chicago, IL, USA) was used for statistical analysis of the data, and the MeV (Multiple Experiment Viewer) was used for heatmap analysis. The differences in mean were calculated using the Duncan’s multiple-range tests for means with 95% confidence limit (*p* < 0.05). Principal components analysis was used to analyze relationships between compounds concentration and their bioactivities.

## 4. Conclusions

In this study, the constituent, antioxidant and anti-proliferative effects of the extracts from three different processed garlics (GG, BG and LAG) and the untreated garlic (WG) were compared. The results indicated that the processed methods significantly affected composition, and the contents of the total sugar, total protein, total phenolics and total flavonoids increased in different degrees after processing. The contents of the above ingredients in BG were the highest compared with the other three garlics. Except for black garlic, where the organosulfur compounds in GG and LAG increased compared with WG. Though BG had a lower score on the PCA, BG and LAG had the highest antioxidant and antiproliferative activity, and reduced the strongest pungency. Taken together, after the comparison and analysis of the related components, the antioxidant and antiproliferation effects of four garlic varieties, BG, GG and LAG are better candidates for related functional food than WG. The results can provide some helpful information for the processing of garlic products.

## Figures and Tables

**Table 1:** The chemical constituents contents of water-soluble extracts and lipid-soluble extracts of WG, GG, BG and LAG.

Garlic Product	WG	GG	BG	LAG
Water-soluble extracts				
Total sugar (mg/g)	8.67 ± 0.04 ^a^	10.61 ± 0.22 ^b^	16.80 ± 0.74 ^c^	7.89 ± 0.96 ^a^
Uronic acid (mg/g)	1.73 ± 0.15 ^a^	2.28 ± 0.06 ^c^	3.91 ± 0.08 ^c^	1.70 ± 0.15 ^a^
Protein (%)	60.03 ± 1.52 ^a^	65.21 ± 1.15 ^b^	77.32 ± 1.24 ^c^	62.12 ± 2.11 ^a^
Monosaccharide composition (mol%)				
Fructose	11.23	12.59	5.70	4.35
Arabinose	2.67	2.28	14.24	6.52
Xylose	5.88	2.08	4.54	5.98
Mannose	19.25	0.39	2.39	1.63
Galactose	7.49	72.75	65.43	27.17
Glucose	53.48	9.91	7.70	54.35
Lipid-soluble extracts				
Organosulfur compounds content (%)	1.54 ± 0.07 ^a^	2.41 ± 0.14 ^c^	0.89 ± 0.14 ^a^	1.83 ± 0.06 ^a^
Total phenolics content (mg GAE/g extracts)	1.25 ± 0.11 ^a^	1.38 ± 0.07 ^a^	13.62 ± 0.91 ^c^	1.56 ± 0.07 ^a^
Total flavonoids content (mg QE/g extracts)	0.32 ± 0.03 ^a^	0.34 ± 0.04 ^a^	0.74 ± 0.08 ^c^	0.45 ± 0.06 ^a^

^a,b,c^ Means with superscripts having different letters in the same line are significantly (*p* < 0.05) different. WG: white garlic; GG: green garlic; BG: black garlic; and LAG: Laba garlic.

**Table 2:** GC-MS analysis of the constituents in the n-hexane extracts of WG, GG, BG and LAG.

	Rt. Time (min)	Accepted Identification	Molecular Weight (Da)	Principal Fragments (*m*/*z*)	Formula	Relative Area (%)			
						WG	GG	BG	LAG
1	4.95	Diallyl disulfide	146	148, 146, 113, 81, 73, 45	C_6_H_10_S_2_	0.92	2.36	1.60	1.77
2	5.113	2-Hexylthiophene	168	168, 111, 97, 69, 45	C_10_H_16_S	-	1.81	0.84	1.21
3	5.461	Allyl methyl sulfide	88	88, 73, 61, 45	C_4_H_8_S	0.01	1.05	-	1.40
4	5.650	1,3,5-Trithiane	138	138, 92, 73, 64, 46	C_3_H_6_S_3_	-	-	3.21	-
5	5.718	Allyl methyl trisulfide	152	152, 114, 105, 87, 79, 73, 45	C_4_H_8_S_3_	0.01	6.58	10.76	10.24
6	5.810	2-tert-Butylhiophenol	166	166, 151, 123, 109, 91, 69, 45	C_10_H_14_S	-	9.06	12.43	9.40
7	6.230	Butylthiobenzene	166	166, 123, 110, 91, 77, 51	C_10_H_14_S	-	1.05	0.25	-
8	6.875	1-Propene-1,1′-thiobis	114	114, 99, 85, 59, 45	C_6_H_10_S	0.63	3.82	0.18	3.65
9	6.695	Diallyl trisulfide	178	178, 113, 73, 47, 45	C_6_H_10_S_3_	0.58	4.03	0.16	6.10
10	7.287	3-Acetyl-2,6-heptanedione	170	170, 128, 95, 85, 71, 58	C_9_H_14_O_3_	-	1.26	1.30	0.93
11	8.003	Ethyl-4-t-butylbenziate	206	206, 191, 163, 115, 107, 91, 77	C_13_H_18_O_2_	6.00	-	12.70	3.03
12	8.328	Diallyl tetrasulphide	210	210, 146, 105, 73, 64	C_6_H_10_S_4_	0.03	2.52	-	1.57
13	8.978	2,5-Dimethyl thiophene	112	112, 111, 97, 77, 59, 45	C_6_H_8_S	-	-	-	0.46
14	10.404	Acetyl valeryl	128	128, 113, 99, 85, 71, 57	C_7_H_12_O_2_	1.07	1.42	-	-
15	10.690	1,2-Dithiin-3-ethenyl-3,6-dihydro	144	144, 111, 103, 97, 85, 77, 71	C_6_H_8_S_2_	-	4.03	-	1.38
16	12.097	10-Nonadecanol	266	266, 157, 125, 111, 97, 83, 69, 55	C_19_H_40_O	0.78	1.01	-	-
17	12.612	Ethyl hexadecanoate	284	284, 239, 157, 101, 88, 73, 55	C_18_H_36_O_2_	-	1.52	-	0.58
18	13.676	(Z, Z, Z)-9, 12, 15-Octade-catrienoyl ethyl ester	306	306, 261, 250, 191, 149, 135, 121, 108, 95, 79, 67, 55	C_20_H_34_O_2_	-	0.80	-	1.93

WG: white garlic; GG: green garlic; BG: black garlic; LAG: Laba garlic.

**Table 3:** The inhibition rate of WG, GG, BG and LAG on HeLa cell; SKOV3 cell; and SMMC-7721 cell.

Cell Line	Concentration (μg·mL^−1^)	WG	GG	BG	LAG
		Cell Growth Inhibition (%)			
HeLa	20	15.84 ± 1.17 ^a^	10.76 ± 0.56 ^a^	25.89 ± 5.22 ^b^	14.24 ± 1.36 ^a^
	40	31.20 ± 0.86 ^a^	29.99 ± 1.422 ^a^	41.79 ± 1.27 ^b^	31.83 ± 2.40 ^a^
	60	41.35 ± 1.43 ^b^	46.67 ± 2.65 ^c^	52.69 ± 3.26 ^d^	35.84 ± 2.18 ^a^
	80	55.99 ± 2.19 ^b^	51.27 ± 1.77 ^b^	55.06 ± 2.66 ^b^	44.723 ± 2.98 ^a^
	100	59.97 ± 1.11 ^a,b^	64.81 ± 3.32 ^b^	60.32 ± 3.49 ^a,b^	55.95 ± 1.14 ^a^
IC_50_ (μg·mL^−1^)		72.51 ± 3.00 ^b^	70.40 ± 3.95 ^b^	60.03 ± 6.79 ^a^	88.57 ± 3.81 ^c^
SKOV3	20	5.98 ± 1.10 ^b^	3.51 ± 0.88 ^a^	3.49 ± 0.65 ^a^	4.80 ± 0.45 ^a,b^
	40	15.07 ± 0.64 ^a^	15.40 ± 0.98 ^a^	29.82 ± 4.18 ^c^	22.23 ± 1.87 ^b^
	60	38.40 ± 2.50 ^a^	38.97 ± 3.51 ^a^	40.37 ± 4.36 ^a^	36.07 ± 1.67 ^a^
	80	56.72 ± 4.10 ^b^	51.65 ± 1.19 ^a,b^	51.53 ± 2.73 ^a,b^	49.13 ± 4.10 ^a^
	100	67.13 ± 5.19 ^b^	54.51 ± 2.35 ^a^	55.45 ± 3.81 ^a^	58.31 ± 2.11 ^a^
IC_50_ (μg·mL^−1^)		74.04 ± 4.50 ^a^	82.56 ± 2.87 ^b^	78.83 ± 2.86 ^a,b^	81.80 ± 4.40 ^b^
SMMC-7721	20	13.69 ± 2.33 ^a^	19.60 ± 3.65 ^a^	36.67 ± 3.01 ^b^	38.93 ± 3.78 ^b^
	40	28.9 ± 3.37 ^a^	35.45 ± 6.14 ^a^	50.23 ± 3.27 ^b^	52.71 ± 0.46 ^b^
	60	37.45 ± 2.65 ^a^	52.12 ± 2.12 ^b^	60.48 ± 2.15 ^c^	84.49 ± 1.83 ^d^
	80	47.43 ± 1.14 ^a^	55.35 ± 3.25 ^b^	68.99 ± 2.57 ^c^	85.88 ± 2.18 ^d^
	100	63.41 ± 2.72 ^a^	63.13 ± 2.12 ^a^	84.33 ± 4.10 ^b^	86.74 ± 4.32 ^b^
IC_50_ (μg·mL^−1^)		78.03 ± 4.91 ^c^	63.76 ± 4.80 ^b^	28.82 ± 1.53 ^a^	35.63 ± 1.97 ^a^

^a,b,c,d^ Means with superscripts having different letters in the same line are significantly (*p* < 0.05) different. WG: white garlic; GG: green garlic; BG: black garlic; LAG: Laba garlic; Hela: human cervical cancer cells; SKOV3: human ovarian cancer cells; SMMC-7721: hepatocellular carcinoma cells; and IC_50_: the half maximal inhibitory concentration.

**Table 4:** Rotated component matrix by the principal component analysis.

	Components		
	1	2	3
TP	−0.975	0.180	0.133
TF	−0.933	0.351	−0.083
OSC	0.925	0.267	0.271
UA	−0.8892	0.258	0.370
SP (total protein)	−0.879	0.363	0.310
TC (total sugar)	−0.887	0.204	0.413
Diallyl disulfide	0.213	−0.976	−0.048
2-Hexylthiophene	0.311	0.885	0.346
Allyl methyl sulfide	0.666	0.705	−0.243
1,3,5-trithiane	−0.976	0.162	0.144
Allyl methyl trisulfide	−0.412	0.892	−0.188
2-tert-butylhiophenol	−0.436	0.895	0.095
Butythiobenzene	0.299	0.415	0.859
1-propene-1,1′-thiobis	0.770	0.637	−0.025
Diallyl trisulfide	0.672	0.655	−0.347
3-Acetyl-2,6-heptanedione	−0.285	0.901	0.326
Ethyl 4-t-butylbenziate	−0.968	−0.223	−0.114
Diallyl tetrasulphide	0.716	0.646	0.265
2,5-dimethyl thiophene	0.296	0.465	−0.834
Acetyl valeryl	0.620	−0.374	0.690
1,2-Dithiin-3-ethenyl-3,6-dihydro	0.654	0.545	0.524
10-nonadecanol	0.618	−0.389	0.683
Ethyl hexadecanoate	0.666	0.563	0.489
(Z, Z, Z)-9, 12, 15-Octade-catrienoyl ethyl ester	0.538	0.647	−0.540
DPPH	0.909	−0.377	−0.180
DR (hydroxyl radical)	0.641	−0.736	0.216
Hela	0.671	0.199	−0.714
SKOV3	0.261	0.959	0.112
SMMC-7721	0.606	−0.705	0.368

TP: total phenolics; TF: total flavonoids; OSC: organosulfur compounds; UA: uronic acid; SP: total protein; TC: total sugar; DPPH: 1,1′-diphenyl-2-picrylhydrazyl; DR: hydroxyl radical; HeLa: human cervical cancer cells; SKOV3: human ovarian cancer cells; and SMMC-7721: hepatocellular carcinoma cells.

## Data Availability

Not applicable.

## Abbreviations

White garlic (WG), green garlic (GG), Laba garlic (LAG), black garlic (BG), gas chromatography-mass spectrometry (GC-MS), 1,1′-diphenyl-2-picrylhydrazyl (DPPH), 3-(4,5-dimethylthiazol-2-yl)-2,5-diphenyl tetrazolium bromide (MTT), principal components analysis (PCA), the half maximal inhibitory concentration (IC_50_), hepatocellular carcinoma cells (SMMC-7721), diallyl sulfide (DAS), diallyl disulfide (DATS), S-allyl cysteine (SAC), dried weight (DW), gallic acid equivalents (GAE), quercetin equivalents (QE), γ-glutamyl-*S*-allyl-L-cysteine (GSAC), human cervical cancer cells (HeLa), human ovarian cancer cells (SKOV3), diallyl disulfide (DADS), uronic acid (UA), principal component 1 (PC-1), principal component 2 (PC-2), principal component 3 (PC-3), principal component (PC), total phenolics (TP), total flavonoids (TF), organosulfur compounds (OSC), allyl methyl trisulfide (AMTS), total protein (SP), total sugar (TC), hydroxyl radical (DR), Folin–Ciocalteu reagent (FC), gas chromatography (GC), total phenolics contents (TPC), total flavonoids contents (TFC), butyl hydroxyanisole (BHA), Dulbecco’s Modified Eagle Medium (DMEM medium), dimethyl sulfoxide (DMSO), standard deviation (SD), Multiple Experiment Viewer (MeV), Diallyl disulfide (G1), 2-Hexylthiophene (G2), Allyl methyl sulfide (G3), 1,3,5-trithiane (G4), Allyl-methyl trisulfide (G5), 2-tert-butylhiophenol (G6), Butythiobenzene (G7), 1-propene-1,1′-thiobis (G9), Diallyl trisulfide (G9), 3-Acetyl-2,6-heptanedione (G10), Ethyl 4-t-butylbenziate (G11), Diallyl tetrasulphide (G12), 2,5-dimethyl thiophene (G13), Acetyl valeryl (G14), 1,2-Dithiin-3-ethenyl-3,6-dihydro (G15), 10-nonadecanol (G16), Ethyl hexadecanoate (G17) and (Z, Z, Z)-9, 12, 15-Octade-catrienoyl ethyl ester (G18).
